# Supplementary material for: Mannose receptor‐derived peptides neutralize pore‐forming toxins and reduce inflammation and development of pneumococcal disease
Source: EMBO Mol Med. 2020 Sep 28;12(11):e12695. doi: 10.15252/emmm.202012695 (PMC7645366; doi:10.15252/emmm.202012695)
Supplement: Supplementary file 1 — Appendix [file EMMM-12-e12695-s001.pdf]

Appendix

Mannose receptor-derived peptides neutralize pore-forming toxins and reduce inflammation and development of pneumococcal disease

Karthik Subramanian[1], Federico Iovino[1], Vasiliki Tsikourkitoudi[1], Padryk Merkl[1], Sultan Ahmed[2], Samuel B. Berry[3], Marie-Stephanie Achtgen[1], Mattias Svensson[3], Peter Bergman[2,4], Georgios A. Sotiriou[1], Birgitta Henriques-Normark[1,5,6#]

[1]Department of Microbiology, Tumor and Cell Biology, Karolinska Institutet, SE-171 77 Stockholm, Sweden.

[2] Department of Laboratory Medicine, Karolinska Institutet, Huddinge, Sweden.

[3]Center for Infectious Medicine, Department of Medicine, Karolinska Institutet, Karolinska University Hospital, Huddinge, Sweden.

[4]Department of Infectious Diseases, the Immunodeficiency Unit, Karolinska University Hospital, Huddinge, Stockholm, Sweden

[5]Clinical Microbiology, Karolinska University Hospital, SE-171 76 Stockholm, Sweden.

[6]Lee Kong Chian School of Medicine (LKC) and Singapore Centre on Environmental Life Sciences Engineering (SCELSE), Nanyang Technological University, Singapore 639798, Singapore.

[#] Correspondence to Birgitta Henriques-Normark (birgitta.henriques@ki.se)

Content

Appendix Figure S1. Intracellular bacterial localization in infected DCs upon treatment with MRC-1 peptides.

Appendix Figure S2. Characterization of peptide-loaded CaP NPs and testing of lung delivery and toxicity *in vivo*.

Appendix Table S1. Sequence and domain location of MRC-1 peptides.

Appendix Table S2. MRC-1 amino acid residues that formed hydrogen bonding interactions with loop residues in the toxins, PLY, LLO and SLO.

Appendix Table S3. ED50 values of peptides P2 and P3 vs the purified CDC toxins.

Appendix Table S4. Exact P values and statistical tests used for the figures

Appendix Materials and Methods

Appendix Figure S1. **Intracellular bacterial localization in infected DCs upon treatment with MRC-1 peptides.** (**A**) Quantification of percentage of intracellular *S. pyogenes* (n=50) in infected DCs that co-localize with MRC-1 and LC3B. Data are mean±s.e.m from two independent experiments. **** denotes P <0.0001 by two-way ANOVA with Bonferroni post hoc test for multiple comarisons. n.s. denotes not significant. **(B)** DCs were infected with *S. pyogenes* (S.py) type M1T1 (left panel) and the isogenic SLO mutant, S. py*Δslo* (right panel) in the presence of 100 μM peptides, P2 or CP2 at MOI of 10 for 2 h. Immunofluorescence microscopy images show that in infected DCs treated with peptide P2 (but not the control peptide CP2), intracellular streptococci (green) do not co-localize with MRC-1 (red), but with the autophagy protein, LC3B (cyan). Peptide P2 had no effect on co-localization of S. py*Δslo* (green) which always co-localized with LC3B (pink), but not with MRC-1(red). Scale bars, 10 μm.

Appendix Figure S1

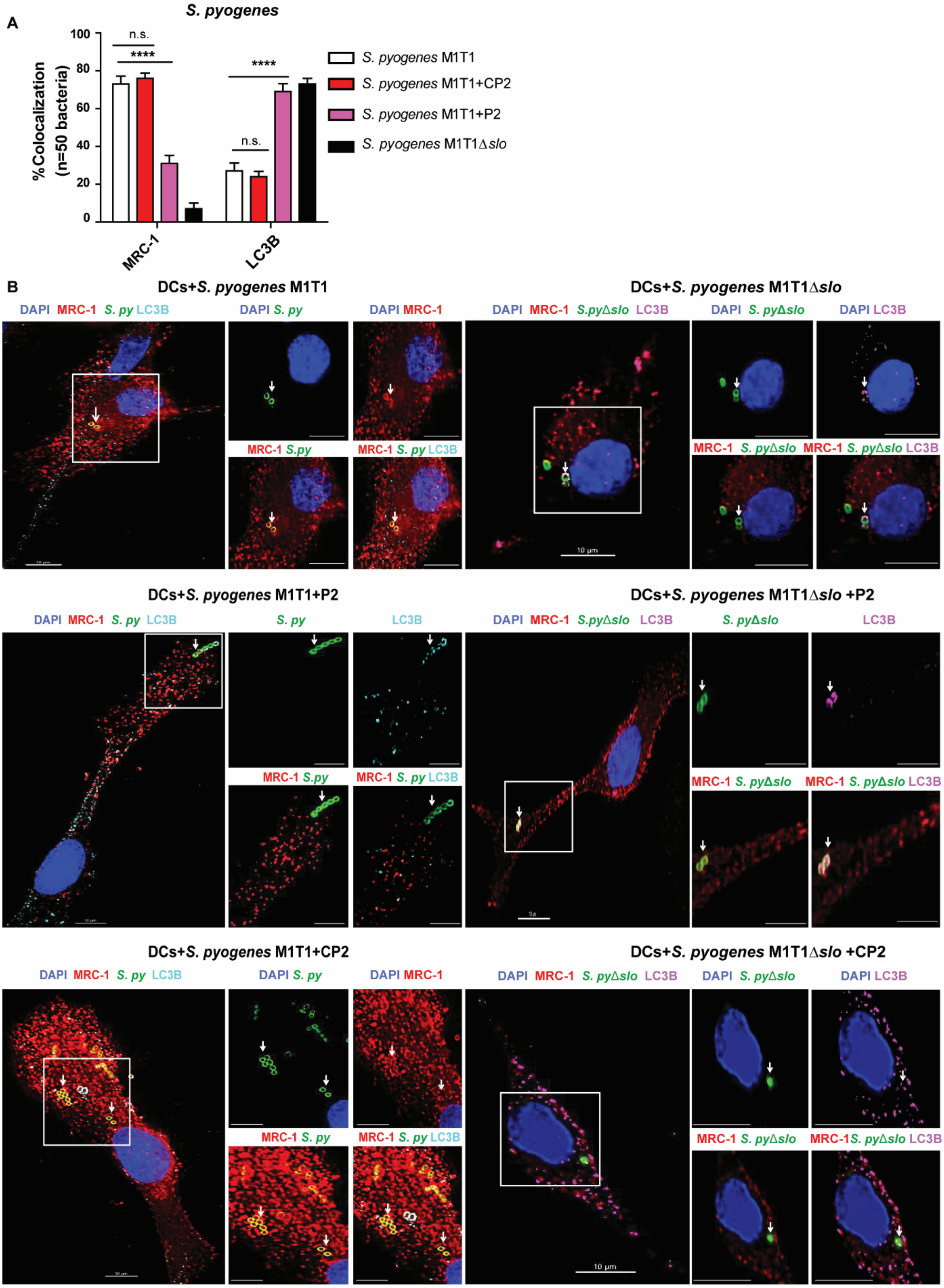

Appendix Figure S2. Characterization of peptide-loaded CaP NPs and testing of lung delivery and toxicity *in vivo*. (A) TEM image of calcium phosphate (CaP) nanoparticles (NPs), exhibiting the characteristic fractal-like agglomerate structure. Scale bar, 50 nm. (**B**) Size distribution of CaP NPs before and after loading with MRC-1 peptide in pure H_2O (solid lines) and PBS (broken lines) was determined by Dynamic light scattering (particle concentration 100 µg/ml). (**C**) Loading capacity of MRC-1 peptides, P2 and CP2 (concentration 100 µg/ml), as a function of NP concentration after overnight co-incubation at room temperature. The loaded amount of MRC-1 peptide is decreased when the particle concentration is increased (constant input peptide concentration 100 µg/ml), probably because of increased agglomeration at higher particle concentrations resulting in a lower available surface area for bioconjugation. (**D**) Dose-dependent inhibition of *S. pneumoniae* T4 induced hemolysis by MRC-1 peptide loaded CaP NPs (peptide content 0-0.5 µg, CaP NPs 0.25-1.25 mg/ml). NPs loaded with the control peptide, CP2, and unloaded NPs served as negative controls. **** denotes $P < 0.0001$ by two-way ANOVA with Dunnett's post-test. (**E**) IVIS imaging showing the lung distribution of NPs loaded with Cy7 tagged peptide P2 (5 µg peptide; 25 µg CaP NPs/mouse) was measured at 1 h and 24 h post intranasal instillation of NPs in mice. Unloaded NPs and Cy7 dye alone served as negative controls. (**F**) Hematoxylin and eosin (H&E) staining of mouse lungs at 24 h post intranasal administration of NPs alone (25 µg CaP NPs/mouse) or P2-NPs (5µg peptide; 25 µg CaP NPs/mouse) or P2 alone (5 µg/mouse). Peptide P2 or P2-NPs were not toxic as revealed by the lung histology analysis which showed that the mice had a normal lung morphology with intact alveolar space (Al) (magnified in the inset) and absence of inflammatory cells. Al-alveolar space; Br-bronchiole; Bv- blood vessel. Scale bars, 50 µm.

Appendix Figure S2

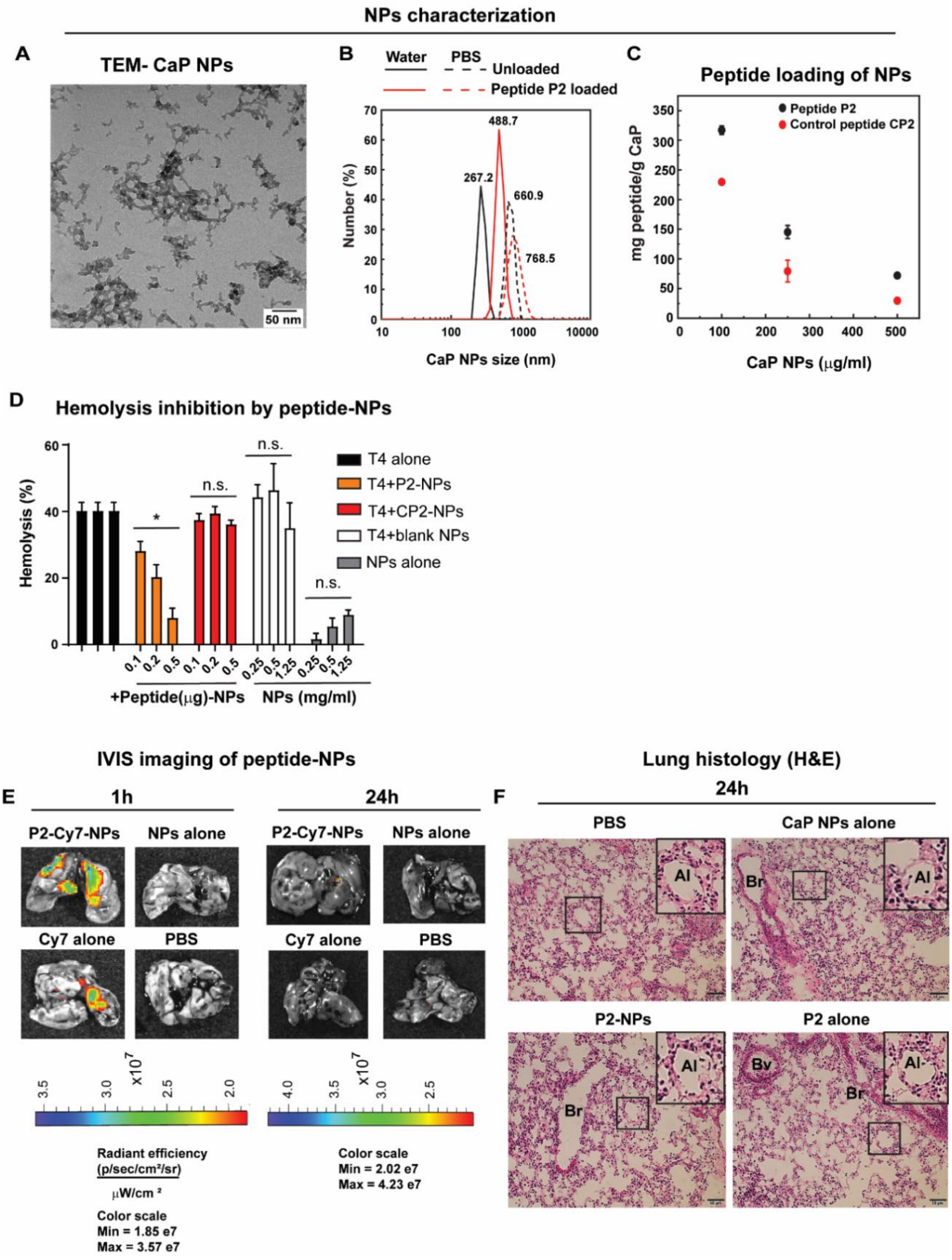

Appendix Table S1. Sequence and domain location of MRC-1 peptides. The residues predicted to form hydrogen bonding interactions with the cholesterol binding loop are highlighted in red.

**Peptide nomenclature**	**Sequence**	**Location on MRC-1**
P1	716-GLTYGSPSEGFTW-728	CTLD4
P2	726-FTWSDGSPVSYEN-738	CTLD4
P3	736-YENWAYGEPNNYQ-748	CTLD4
P4	746-NYQNVEYCGELKG-758	CTLD4
P5	756-LKGDPTMSWNDIN-768	CTLD4
P6	766-DINCEHLNNWICQ-778	CTLD4
CP1	181-DCTSAGRSDGWLW-193	FN II domain
CP2	1444-LVGNIEQNEHSVI-1456	Intracellular tail
Scrambled peptide P2	PDSTFWNGESVYS	

Appendix Table S2: Amino acids predicted to be involved in hydrogen bonding interactions from computational docking. MRC-1 amino acid residues that are predicted to form hydrogen bonding interactions with loop residues in the toxins, PLY, LLO and SLO.

**Protein**	**PLY: MRC1**	**LLO: MRC1**	**SLO: MRC1**
PLY/LLO/SLO	W433, W435, W436, E434	W489, E490	T531
MRC-1	T727, S729, E737, Y719, Y736, N738	S729, Y736, N738	Y719, Y736 Y752, D766

Appendix Table S3. ED50 values of peptides P2 and P3 vs the purified CDC toxins. Median effective dose (ED50) of the peptides P2 and P3 against the purified toxins.

**Peptide ED50 (μM)**	**PLY**	**LLO**	**SLO**
P2	6.6	22.3	9.8
P3	21.9	88.5	17.7

Appendix Table S4. **P values and Statistical tests used**

Figure and comparsion	P value	Statistical test
3B CP2 vs P2	0.0181	One-way ANOVA with Dunnet's post hoc test for multiple comarisons
3B CP2 vs P3	0.0307	One-way ANOVA with Dunnet's post hoc test for multiple comarisons
3B CP2 vs Scrambled P2	0.984165007	One-way ANOVA with Dunnet's post hoc test for multiple comarisons
3C PLY vs PLY+P2	8.09406E-13	Two-way ANOVA with Bonferroni post hoc test for multiple comarisons
3C PLY vs PLY+P3	4.21659E-10	Two-way ANOVA with Bonferroni post hoc test for multiple comarisons
3C PLY vs PLY+Scrambled P2	0.606619555	Two-way ANOVA with Bonferroni post hoc test for multiple comarisons
3C PLY vs PLY+CP2	0.14465728	Two-way ANOVA with Bonferroni post hoc test for multiple comarisons
3C LLO vs LLO+P2	1.15867E-14	Two-way ANOVA with Bonferroni post hoc test for multiple comarisons
3C LLO vs LLO+P3	3.06171E-11	Two-way ANOVA with Bonferroni post hoc test for multiple comarisons
3C LLO vs LLO+Scrambled P2	0.654541975	Two-way ANOVA with Bonferroni post hoc test for multiple comarisons
3C LLO vs LLO+CP2	0.453241024	Two-way ANOVA with Bonferroni post hoc test for multiple comarisons
3C SLO vs SLO+P2	1.89427E-16	Two-way ANOVA with Bonferroni post hoc test for multiple comarisons
3C SLO vs SLO+P3	1.03078E-14	Two-way ANOVA with Bonferroni post hoc test for multiple comarisons
3C SLO vs SLO+Scrambled P2	0.999359055	Two-way ANOVA with Bonferroni post hoc test for multiple comarisons

3C SLO vs SLO+CP2	0.472294596	Two-way ANOVA with Bonferroni post hoc test for multiple comarisons
3E T4 vs T4+P2	0.0006	One-way ANOVA Bonferroni post hoc test for multiple comarisons
3E T4 vs T4+CP2	0.2300	One-way ANOVA Bonferroni post hoc test for multiple comarisons
4A PLY vs PLY+P2	5.65613E-15	Two-way ANOVA with Bonferroni post hoc test for multiple comarisons
4A PLY vs PLY+P3	2.54751E-06	Two-way ANOVA with Bonferroni post hoc test for multiple comarisons
4A PLY vs PLY+CP2	0.416488068	Two-way ANOVA with Bonferroni post hoc test for multiple comarisons
4A LLO vs LLO+P2	9.4799E-14	Two-way ANOVA with Bonferroni post hoc test for multiple comarisons
4A LLO vs LLO+ P3	9.95856E-14	Two-way ANOVA with Bonferroni post hoc test for multiple comarisons
4A LLO vs LLO+CP2	0.100251389	Two-way ANOVA with Bonferroni post hoc test for multiple comarisons
4A SLO vs SLO+P2	2.9997E-09	Two-way ANOVA with Bonferroni post hoc test for multiple comarisons
4A SLO vs SLO+P3	2.18237E-11	Two-way ANOVA with Bonferroni post hoc test for multiple comarisons
4A SLO vs SLO+CP2	0.748488825	Two-way ANOVA with Bonferroni post hoc test for multiple comarisons
4D T4 vs T4+P2	0.005	One-way ANOVA with Dunnet's post hoc test for multiple comarisons
4D T4 vs T4+CP2	0.4236	One-way ANOVA with Dunnet's post hoc test for multiple comarisons
4D T4 vs T4Δ*ply*	0.0038	One-way ANOVA with Dunnet's post hoc test for multiple comarisons
4D T4 vs T4+a-Ply	0.0023	One-way ANOVA with Dunnet's post hoc test for multiple comarisons

4E T4 vs T4+P2	1.31398E-08	Two-way ANOVA with Bonferroni post hoc test for multiple comarisons
4E T4 vs T4+CP2	0.369710844	Two-way ANOVA with Bonferroni post hoc test for multiple comarisons
4E T4 vs T4+a-Ply	2.7207E-09	Two-way ANOVA with Bonferroni post hoc test for multiple comarisons
4E D39 vs D39+P2	2.92232E-11	Two-way ANOVA with Bonferroni post hoc test for multiple comarisons
4E D39 vs D39+CP2	0.138990721	Two-way ANOVA with Bonferroni post hoc test for multiple comarisons
4E D39 vs D39+a-Ply	1.16504E-12	Two-way ANOVA with Bonferroni post hoc test for multiple comarisons
4G MRC1: T4R vs T4R+P2	7.1395E-06	Two-way ANOVA with Bonferroni post hoc test for multiple comarisons
4G MRC1: T4R vs T4R+CP2	0.46323912	Two-way ANOVA with Bonferroni post hoc test for multiple comarisons
4G LC3B: T4R vs T4R+P2	7.1395E-06	Two-way ANOVA with Bonferroni post hoc test for multiple comarisons
4G LC3B: T4R vs T4R+CP2	0.46323912	Two-way ANOVA with Bonferroni post hoc test for multiple comarisons
5A T4 vs. T4Δply	1.21821E-09	Log-rank (Mantel-Cox)
5A Mock vs T4	5.79512E-15	Log-rank (Mantel-Cox)
5B T4 vs T4+P2-NPs	2.59477E-07	Log-rank (Mantel-Cox)
5B T4 vs T4+P2	0.0002	Log-rank (Mantel-Cox)
5B T4+P2 vs T4+CP2	5.21034E-05	Log-rank (Mantel-Cox)
5B TP2-NPs vs T4+NPs	4.7568E-07	Log-rank (Mantel-Cox)
5B T4 vs T4-NPs	0.6140	Log-rank (Mantel-Cox)

5C T4 vs. T4Δply	0.0043	Log-rank (Mantel-Cox)
5C T4 vs. T4+P2	0.0471	Log-rank (Mantel-Cox)
5C T4 vs. T4+P2-NPs	0.0014	Log-rank (Mantel-Cox)
5C T4+NPs vs. T4+P2- NPs	0.0343	Log-rank (Mantel-Cox)
5C T4+P2 vs. T4+P2-NPs	0.0427	Log-rank (Mantel-Cox)
5D T4 vs. T4Δply	1.14992E-09	One-way ANOVA Bonferroni post hoc test for multiple comarisons
5D T4 vs. T4+P2	1.15013E-10	One-way ANOVA Bonferroni post hoc test for multiple comarisons
5D T4 vs. T4+P2-NPs	1.01609E-14	One-way ANOVA Bonferroni post hoc test for multiple comarisons
5D T4 vs. T4+NPs alone	0.1752	One-way ANOVA Bonferroni post hoc test for multiple comarisons
5D T4+P2 vs. T4+P2-NPs	0.0489	One-way ANOVA Bonferroni post hoc test for multiple comarisons
5D T4+P2-NPs vs. T4+NPs alone	2.25685E-11	One-way ANOVA Bonferroni post hoc test for multiple comarisons
5E T4 vs. T4Δply	1.04894E-05	One-way ANOVA Bonferroni post hoc test for multiple comarisons
5E T4 vs. T4+P2	0.0032	One-way ANOVA Bonferroni post hoc test for multiple comarisons
5E T4 vs. T4+P2-NPs	2.03826E-09	One-way ANOVA Bonferroni post hoc test for multiple comarisons
5E T4 vs. T4+NPs alone	0.626277135	One-way ANOVA Bonferroni post hoc test for multiple comarisons
5E T4+P2 vs. T4+P2-NPs	0.0255	One-way ANOVA Bonferroni post hoc test for multiple comarisons
5E T4+P2-NPs vs. T4+NPs alone	5.21004E-08	One-way ANOVA Bonferroni post hoc test for multiple comarisons

5F T4 vs. T4Δply	4.24476E-07	One-way ANOVA Bonferroni post hoc test for multiple comarisons
5F T4 vs. T4+P2	0.005	One-way ANOVA Bonferroni post hoc test for multiple comarisons
5F T4 vs. T4+P2-NPs	2.2348E-07	One-way ANOVA Bonferroni post hoc test for multiple comarisons
5F T4 vs. T4+NPs alone	0.7299	One-way ANOVA Bonferroni post hoc test for multiple comarisons
5F T4+P2 vs. T4+P2-NPs	0.037236595	One-way ANOVA Bonferroni post hoc test for multiple comarisons
5F T4+P2-NPs vs. T4+NPs alone	3.07575E-06	One-way ANOVA Bonferroni post hoc test for multiple comarisons
EV1F PLY (no blockade vs blockade)	0.0073	Two-way ANOVA with Bonferroni post hoc test for multiple comarisons
EV1F LLO (no blockade vs blockade)	0.0096	Two-way ANOVA with Bonferroni post hoc test for multiple comarisons
EV1F SLO (no blockade vs blockade)	0.0101	Two-way ANOVA with Bonferroni post hoc test for multiple comarisons
EV2C PLY+P2 vs PLY+P4	0.0157	One-way ANOVA Tukey's post hoc test for multiple comarisons
EV2C PLY+P2 vs PLY+P5	0.0172	One-way ANOVA Tukey's post hoc test for multiple comarisons
EV2C PLY+P2 vs PLY+P6	0.0016	One-way ANOVA Tukey's post hoc test for multiple comarisons
EV2C PLY+P3 vs PLY+P4	0.0274	One-way ANOVA Tukey's post hoc test for multiple comarisons
EV2C PLY+P3 vs PLY+P5	0.0302	One-way ANOVA Tukey's post hoc test for multiple comarisons
EV2C PLY+P3 vs PLY+P6	0.0023	One-way ANOVA Tukey's post hoc test for multiple comarisons
EV2C PLY+CP1 vs PLY alone	0.1744	One-way ANOVA Tukey's post hoc test for multiple comarisons

EV2C PLY+CP2 vs PLY alone	0.1088	One-way ANOVA Tukey's post hoc test for multiple comarisons
EV2C PLY+BSA vs PLY alone	0.9968	One-way ANOVA Tukey's post hoc test for multiple comarisons
EV2C PLY+Cholesterol vs PLY alone	0.0005	One-way ANOVA Tukey's post hoc test for multiple comarisons
EV3C P2+LLO vs CP2+LLO	0.0130	One-way ANOVA with Dunnet's post hoc test for multiple comarisons
EV3C P3+LLO vs CP2+LLO	0.0161	One-way ANOVA with Dunnet's post hoc test for multiple comarisons
EV3C Scrambled P2+LLO vs CP2+LLO	0.8758	One-way ANOVA with Dunnet's post hoc test for multiple comarisons
EV3D P2+SLO vs CP2+SLO	0.0313	One-way ANOVA with Dunnet's post hoc test for multiple comarisons
EV3D P3+SLO vs CP2+SLO	0.0227	One-way ANOVA with Dunnet's post hoc test for multiple comarisons
EV3D Scrambled P2+SLO vs CP2+SLO	0.9987	One-way ANOVA with Dunnet's post hoc test for multiple comarisons
EV3E *S.pyogenes* vs *S.pyogenes*+P2	0.0015	One-way ANOVA Bonferroni post hoc test for multiple comarisons
EV3E *S.pyogenes* vs *S.pyogenes*+CP2	0.3726	One-way ANOVA Bonferroni post hoc test for multiple comarisons
EV3F *L.moncytogenes* vs *L.monocytogenes*+P2	0.0001	One-way ANOVA Bonferroni post hoc test for multiple comarisons
EV3F *L.moncytogenes*+P2 vs *L.monocytogenes*+CP2	0.8552	One-way ANOVA Bonferroni post hoc test for multiple comarisons
EV4A PLY vs PLY+P2	1.67846E-09	Two-way ANOVA with Bonferroni post hoc test for multiple comarisons
EV4A PLY vs PLY+P3	3.33822E-10	Two-way ANOVA with Bonferroni post hoc test for multiple comarisons
EV4A PLY vs PLY+CP2	0.5666	Two-way ANOVA with Bonferroni post hoc test for multiple comarisons

EV4A LLO vs LLO+P2	7.95176E-10	Two-way ANOVA with Bonferroni post hoc test for multiple comarisons
EV4A LLO vs LLO+P3	1.80659E-10	Two-way ANOVA with Bonferroni post hoc test for multiple comarisons
EV4A LLO vs LLO+CP2	0.7046	Two-way ANOVA with Bonferroni post hoc test for multiple comarisons
EV4A SLO vs SLO+P2	3.64372E-09	Two-way ANOVA with Bonferroni post hoc test for multiple comarisons
EV4A SLO vs SLO+P3	6.89615E-08	Two-way ANOVA with Bonferroni post hoc test for multiple comarisons
EV4A SLO vs SLO+CP2	0.9749	Two-way ANOVA with Bonferroni post hoc test for multiple comarisons
EV4B PLY vs PLY+P2	1.15489E-05	Two-way ANOVA with Bonferroni post hoc test for multiple comarisons
EV4B PLY vs PLY+P3	5.46774E-07	Two-way ANOVA with Bonferroni post hoc test for multiple comarisons
EV4B PLY vs PLY+CP2	0.521864243	Two-way ANOVA with Bonferroni post hoc test for multiple comarisons
EV4B LLO vs LLO+P2	0.00150012	Two-way ANOVA with Bonferroni post hoc test for multiple comarisons
EV4B LLO vs LLO+P3	0.001157688	Two-way ANOVA with Bonferroni post hoc test for multiple comarisons
EV4B LLO vs LLO+CP2	0.735384411	Two-way ANOVA with Bonferroni post hoc test for multiple comarisons
EV4B SLO vs SLO+P2	1.24292E-05	Two-way ANOVA with Bonferroni post hoc test for multiple comarisons
EV4B SLO vs SLO+P3	4.50272E-05	Two-way ANOVA with Bonferroni post hoc test for multiple comarisons
EV4B SLO vs SLO+CP2	0.848821137	Two-way ANOVA with Bonferroni post hoc test for multiple comarisons
EV4C PLY vs PLY+P2	0.0189	Paired T test
EV4C PLY vs PLY+CP2	0.5387	Paired T test

EV4D Unt vs PLY	7.35326E-09	One-way ANOVA with Dunnett's post hoc test for multiple comparisons
EV4D PLY vs PLY+P2	7.46644E-07	One-way ANOVA with Dunnett's post hoc test for multiple comparisons
EV4D PLY vs PLY+CP2	0.0560	One-way ANOVA with Dunnett's post hoc test for multiple comparisons
EV4E Unt vs PLY	1.27056E-06	One-way ANOVA with Dunnett's post hoc test for multiple comparisons
EV4E PLY vs PLY+P2	4.86585E-06	One-way ANOVA with Dunnett's post hoc test for multiple comparisons
EV4E PLY vs PLY+CP2	0.1586	One-way ANOVA with Dunnett's post hoc test for multiple comparisons
EV4F Unt vs PLY	0.000315571	One-way ANOVA with Dunnett's post hoc test for multiple comparisons
EV4F PLY vs PLY+P2	0.00195764	One-way ANOVA with Dunnett's post hoc test for multiple comparisons
EV4F PLY vs PLY+CP2	0.952107006	One-way ANOVA with Dunnett's post hoc test for multiple comparisons
Appendix Fig. S1A MRC1 *S.pyogenes* vs *S.pyogenes*+P2	5.21997E-06	Two-way ANOVA with Bonferroni post hoc test for multiple comarisons
Appendix Fig. S1A MRC1 *S.pyogenes* vs *S.pyogenes*+CP2	0.467927807	Two-way ANOVA with Bonferroni post hoc test for multiple comarisons
Appendix Fig. S1A LC3B *S.pyogenes* vs *S.pyogenes*+P2	5.21997E-06	Two-way ANOVA with Bonferroni post hoc test for multiple comarisons
Appendix Fig. S1A LC3B *S.pyogenes* vs *S.pyogenes*+CP2	0.467927807	Two-way ANOVA with Bonferroni post hoc test for multiple comarisons
Appendix Fig. S2D T4+P2-NPs 0.1μg vs 0.5 μg	2.92536E-06	Two-way ANOVA with Dunnet's post hoc test for multiple comarisons
Appendix Fig. S2D T4+CP2-NPs 0.1μg vs 0.5 μg	0.9260	Two-way ANOVA with Dunnet's post hoc test for multiple comarisons
Appendix Fig. S2D T4+blank-NPs 0.1μg vs 0.5 μg	0.1688	Two-way ANOVA with Dunnet's post hoc test for multiple comarisons

Appendix Materials and Methods

Nanoparticle synthesis and characterization

Calcium phosphate (CaP) NPs (d_{BET} = 8 nm) were produced by flame spray pyrolysis as described previously (*1*). The metal-organic precursors calcium acetate hydrate (≥ 99%, Sigma-Aldrich) and europium nitrate hexahydrate (99.9%, Alfa Aesar) were dissolved in a mixture of 2-ethylhexanoic acid (99%, Sigma-Aldrich) and propionic acid (≥ 99.5%, Sigma-Aldrich) in 1:1 ratio and stirred under reflux for 30 min at 70°C. The nanoparticles were doped with Europium to enable their monitoring by luminescence. Subsequently, tributyl phosphate (≥ 99%, Sigma-Aldrich) was added, after a clear solution was observed, in appropriate quantity in order to obtain Ca/P molar ratio of 2.19. The total metal concentration of the precursor solution was 0.1 M. The precursor solution was fed to the FSP nozzle through a capillary tube (SGE Analytical Science) using a syringe pump (New Era Pump Systems, Inc.). The solution was atomized in the FSP nozzle by oxygen gas at 3 L/min (Strandmöllen AB) (EL-FLOW Select, Bronkhorst) at constant pressure drop (1.8 bar). The synthesis of the particles was carried out at 8 ml/min precursor feed flow rate. The spray flame was ignited by a premixed supporting flame of methane/oxygen (Scientific grade, Linde Gas AB) at flow rates of 1.5 L/min and 3.2 L/min, respectively. The particles were collected on a glass fiber filter (Hahnemühle) with the aid of a Mink MM 1144 BV vacuum pump (Busch).

The specific surface area (SSA) was determined by the nitrogen adsorption-desorption isotherms in liquid nitrogen at 77K using a Tristar II Plus (Micromeritics) instrument. The sample was degassed for at least 3 h at 110°C.

The structure of the NPs was observed using transmission electron microscopy (TEM) in a FEI Tecnai BioTWIN instrument operated with an acceleration voltage of 120 kV and equipped with a 2kx2k Veleta OSiS CCD camera. For the TEM imaging, the nanoparticles were suspended in ethanol in a water-cooled cup horn system (VCX750, cup horn Part no. 630-0431, Sonics Vibracell) (10 min, 100% amplitude) and one drop of the suspension was deposited onto a carbon coated copper grid (400 mesh carbon film, S160-4, Agar Scientific). The grid was dried at ambient temperature overnight.

Size distribution of unloaded and MRC1-peptide loaded CaP NPs was evaluated by dynamic light scattering (DLS) with a zetasizer ultra (Malvern Panalytical).

MRC1-Peptide adsorption onto CaP NPs

The MRC1 peptide was loaded onto CaP NPs via physisorption (*2*). Suspensions of MRC1-loaded CaP NPs in PBS pH 7.4 (200 µl sample volume) were prepared by addition of 100 µl of dispersed CaP NPs in PBS pH 7.4 of initial concentration ranging from 200 to 1000 µg/ml to an equal volume of MRC1 peptide solution (initial concentration of 200 µg/ml).The suspensions were placed on a roller mixer (Stuart SRT9D) for gentle mixing at 60 rpm overnight. The particles were separated via centrifugation at 10000 rpm for 20 min and the supernatant containing the unloaded peptide was collected for quantification using a Pierce bicinchoninic acid (BCA) protein assay kit (Thermo Fisher Scientific) according to manufacturer's instructions. Absorbance was measured at 562 nm using a microplate reader (SpectraMax Plus, Molecular Devices) and the amount of peptide was calculated from a calibration curve. The amount of loaded peptide was calculated from the difference between the initial concentration and the concentration of the supernatant. Furthermore, the loaded particles were washed once with PBS and re-dispersed in PBS. The amount of peptide after the washing was also quantified in the supernatant after centrifugation (10000 rpm, 20 min) and was found negligible (≤ 1%) indicating the stability of the conjugates. The final concentration of peptide P2 on the CaP NPs varied between 75-150 mg/g CaP at a NP concentration of 250 µg/ml.

For *in vivo* imaging of the peptide conjugated NPs, we generated fluorophore-loaded peptide NPs by incorporating the near infrared dye, Sulfo-Cy7 amine (Lumiprobe, GmbH) as described previously (*3*). An aqueous solution of the Cy7 (initial concentration 62.5 µg/ml) was co-incubated with MRC-1 peptide loaded NPs overnight on a roller shaker at 60 rpm (Stuart SRT9D). The unconjugated dye was removed by at least 3 washings and centrifugation at 10000 for 15 min. The amount of Cy7 loaded on CaP nanoparticles was measured using an ultraviolet-visible (UV-Vis) spectrophotometer (NanoDrop One, Thermo Scientific) (λ = 750 nm). The concentration of Cy7 was calculated as the difference between the concentration of the initial solution and that of the supernatant. The final concentration of Cy7 within the loaded CaP particles was 29.2 ± 2.24 µg/ml.

Live imaging of cytolysis by bacterial toxins

Human THP-1 monocytes were seeded at $5x10^5$ cells in 12 well plates and differentiated with PMA (20 ng/ml) for 48 h in 12 well plates. Cells were washed with PBS and loaded with live/dead reagent (2 µM Calcein AM and 4 µM Ethidium bromide) for 20 min at 37ºC. The

cell-permeable dye, Calcein AM, becomes green-fluorescent upon hydrolysis by intracellular esterases in live cells, while dead cells are stained red by propidium iodide. Then, 0.5 µg/ml PLY, LLO or SLO with or without 100 µM peptide P2 or control peptide, CP2, was added to the wells. Cholesterol (100 µM) was used as a positive control, while BSA (100 µM) was used as negative control. The plate was mounted on the microscope stage set at 37°C and 5% CO_2 and imaged at 30 second interval for total time of 20 min under the green (488 nm emission) and red (594 nm emission) channels; imaging was performed every 30 seconds to avoid cytolysis induced by the cytotoxic effect of the excitation laser. Images were acquired using a Delta Vision Elite microscope under a 20X objective (GE Healthcare).

Live Imaging of the 3D lung model

After 5 days of air exposure, models were cut out from the Transwell inserts and mounted for live imaging. For mounting, 50 µl of media containing stimulation samples were added to the base of a glass-bottomed well plate (MatTek Corp., Ashland, MA) followed by placement of the separated models apical-side down into the 50 µl to ensure exposure of the model to the sample. A 4% (w/v) low-temp gelling agarose solution was then added around the top of the inverted model (basolateral side) and 1 ml of complete DMEM was added around the outside of the agarose ring to provide nutrients and maintain humidity during the imaging. Once mounted, models were maintained at 37°C and 5% CO_2 and imaged at 5 min intervals starting from 45 min post stimulation until 240 min post stimulation; maximum intensity projections were created for each time point using the Nikon NIS Elements Software (Nikon Inc., Tokyo, Japan), and total GFP expression was then analyzed from each frame over time. Statistical analyses were conducted using Prism (GraphPad Software, San Diego, CA). All images were obtained on a Nikon A1R HD25 confocal microscope at 20X magnification.

Co-localization of bacteria with MRC-1 and LC3B in DCs

Briefly, $2x10^5$ DCs seeded onto coverslips were infected with the unencapsulated type 4 *S. pneumoniae* T4R and its isogenic PLY mutant, T4RΔ*ply,* or the *S. pyogenes* strains M1T1 strain M1T1 and its isogenic SLO mutant at MOI of 10. The MRC-1 peptides, P2 and CP2, were diluted in R10 medium and used at 100 µM. At 2 h post infection, extracellular bacteria were killed by adding gentamicin (200 µg/ml) for 60 min and washed twice with PBS. The DCs were fixed, permeabilized and blocked as described earlier and stained with 1:1000 diluted Alexa 647-conjugated rabbit anti-LC3B antibody (Abcam) overnight. Pneumococci were detected using 1:100 diluted rabbit anti-pneumococcal anti-serum (Eurogentec) labelled with Alexa 488

using a Zenon Rabbit IgG Labeling kit (Thermo Fisher Scientific) for 1h at room temperature. *Streptococcus pyogenes* (Group A streptococci) was detected using 1:50 diluted Alexa 488 conjugated rabbit anti *S. pyogenes* (Abcam) overnight at 4ºC. MRC-1 was detected using Alexa 594-conjugated-Rabbit anti-MRC1 (Abcam). The coverslips were mounted on slides using Prolong Gold anti-fade mounting medium containing the nuclear stain 4,6-diamidino-2-phenylindole (DAPI; Thermo Fisher Scientific). Images were acquired using a Delta Vision Elite microscope under the 100x oil immersion objective (GE Healthcare). The cell boundary was marked by the DC receptor, MRC-1. In some images, LC3B was pseudo-colored to cyan for better color contrast, for quantification of percentage of intracellular bacteria that co-localized with MRC-1 and LC3B.

References cited in the appendix:

1. S. Loher *et al.*, Fluoro-apatite and Calcium Phosphate Nanoparticles by Flame Synthesis. *Chemistry of Materials* **17**, 36-42 (2005).
2. M. Tonigold *et al.*, Pre-adsorption of antibodies enables targeting of nanocarriers despite a biomolecular corona. *Nat Nanotechnol* **13**, 862-869 (2018).
3. M. Miragoli *et al.*, Inhalation of peptide-loaded nanoparticles improves heart failure. *Sci Transl Med* **10**, (2018).
