# Supplementary material for: Mannose receptor‐derived peptides neutralize pore‐forming toxins and reduce inflammation and development of pneumococcal disease
Source: EMBO Mol Med. 2020 Sep 28;12(11):e12695. doi: 10.15252/emmm.202012695 (PMC7645366; doi:10.15252/emmm.202012695)
Supplement: Supplementary file 2 — Expanded View Figures PDF [file EMMM-12-e12695-s002.pdf]

Expanded View Figures

Figure EV1. Co-localization of PLY, LLO, and SLO with MRC-1 and EEA-1 in DCs and effect of MRC-1 blockade.

A Pearson's correlation coefficient showing extent of co-localization of MRC-1 with PLY, LLO, and SLO. Data represent mean ± s.d. from three experiments.

B, C Human DCs were incubated with a non-cytolytic dose (0.2 μg/ml) of purified toxoid derivatives, (B) PLY(W433F) and (C) LLO(W489F) for 45 min. Immunofluorescence staining shows that both PLY(W433F) and LLO(W489F) (green) show weak binding to DCs and do not co-localize with MRC-1 (red) in DCs. Scale bars, 5 μm. Images are representative of three independent experiments.

D, E Human DCs were incubated with 0.2 μg/ml of purified (D) LLO and (E) SLO for 60 min upon pre-treatment with 1 μg/ml of anti-MRC-1. Immunofluorescence microscopy shows that upon MRC-1 blockade, the binding of LLO and SLO (green) to DCs is diminished and do not co-localize with the early endosomal antigen, EEA-1 (purple). Scale bars, 10 μm.

F Pearson's correlation coefficient showing extent of co-localization of EEA-1 with PLY, LLO, and SLO with or without MRC-1 antibody blockade. Upon antibody blockade of MRC-1, the extent of co-localization with EEA-1 was reduced. Data represent mean ± s.d. from three experiments. *$P < 0.05$; **$P < 0.01$ by two-way ANOVA with Bonferroni post-test. Exact *P* values are shown in Appendix Table S4.

© 2020 The Authors

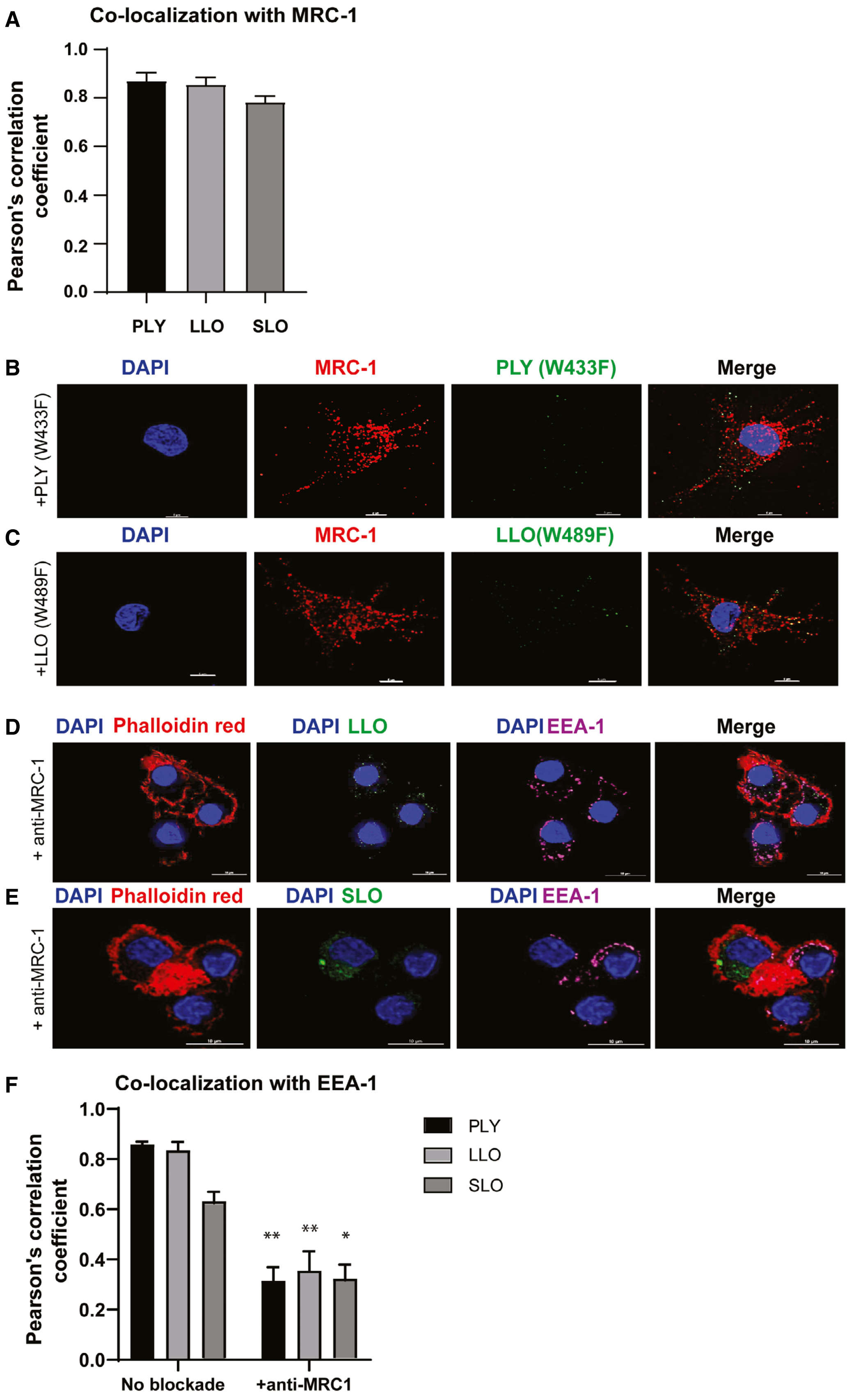
A
Co-localization with MRC-1
Pearson's correlation coefficient
1.0
0.8
0.6
0.4
0.2
0.0
PLY
LLO
SLO
B
DAPI
MRC-1
PLY (W433F)
Merge
+PLY (W433F)
C
DAPI
MRC-1
LLO(W489F)
Merge
+LLO (W489F)
D
DAPI Phalloidin red
DAPI LLO
DAPI EEA-1
Merge
+ anti-MRC-1
E
DAPI Phalloidin red
DAPI SLO
DAPI EEA-1
Merge
+ anti-MRC-1
F
Co-localization with EEA-1
Pearson's correlation coefficient
1.0
0.8
0.6
0.4
0.2
0.0
**
**
*
No blockade
+anti-MRC1
PLY
LLO
SLO

Figure EV1.

Figure EV2. Location and activity of MRC-1 peptides against PLY and LLO.

A Domain architecture and location of peptides (P1–P6, CP1, and CP2) on the MRC-1 protein. P1–P6 are from the CTLD4 domain which binds to the membrane-binding loop of CDCs while the control peptides, CP1 and CP2, are from regions that do not bind CDCs.

B Red blood cell hemolysis assay showing the residual cell pellet after hemolysis by purified PLY and LLO (1 μg/ml) in the presence of 100 μM of MRC-1 peptides. Complete hemolysis indicated by absence of red pellet was achieved by PLY and LLO alone as well as the control peptides, CP1 and CP2, while peptides P1–P6 conferred protection to various extents. Cholesterol was used as positive control to block hemolysis.

C Quantification of hemolysis induced by PLY (1 μg/ml) in the presence of 100 μM of MRC-1 peptides, P1–P6, and control peptides, CP1 and CP2. BSA was used as negative control to show specificity while cholesterol was used as positive control to block hemolysis. Data are mean ± s.e.m. from two experiments with triplicates. **P* < 0.05, ***P* < 0.01, and ****P* < 0.001 by one-way ANOVA with Tukey's *post hoc* test for multiple comparisons. n.s. denotes not significant. Exact *P* values are shown in Appendix Table S4.

 © 2020 The Authors

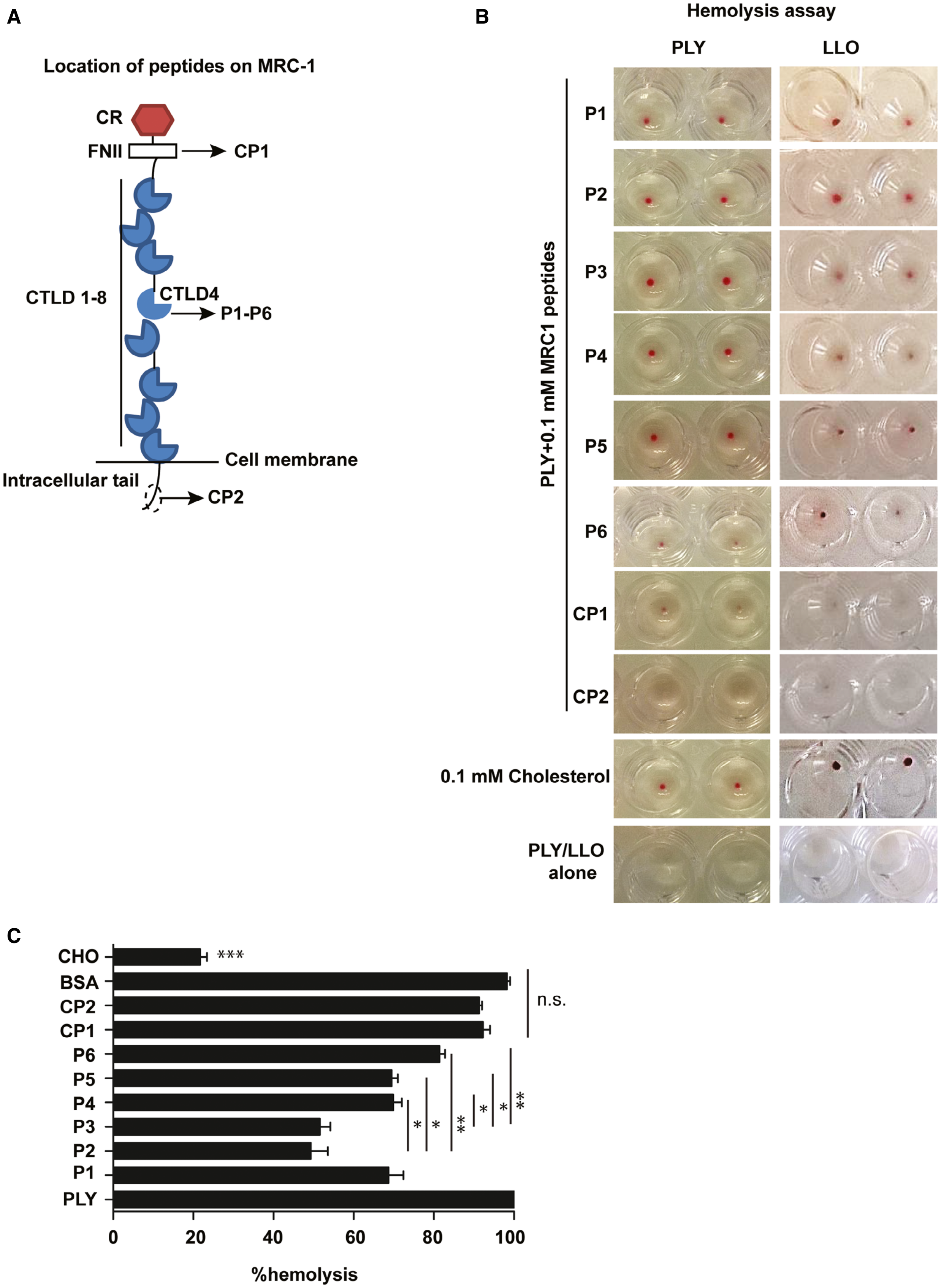
A
Location of peptides on MRC-1
CR
FNII
CP1
CTLD 1-8
CTLD4
P1-P6
Cell membrane
Intracellular tail
CP2
B
Hemolysis assay
PLY
LLO
PLY+0.1 mM MRC1 peptides
P1
P2
P3
P4
P5
P6
CP1
CP2
0.1 mM Cholesterol
PLY/LLO alone
C
CHO
BSA
CP2
CP1
P6
P5
P4
P3
P2
P1
PLY
***
n.s.
0
20
40
60
80
100
%hemolysis

Figure EV2.

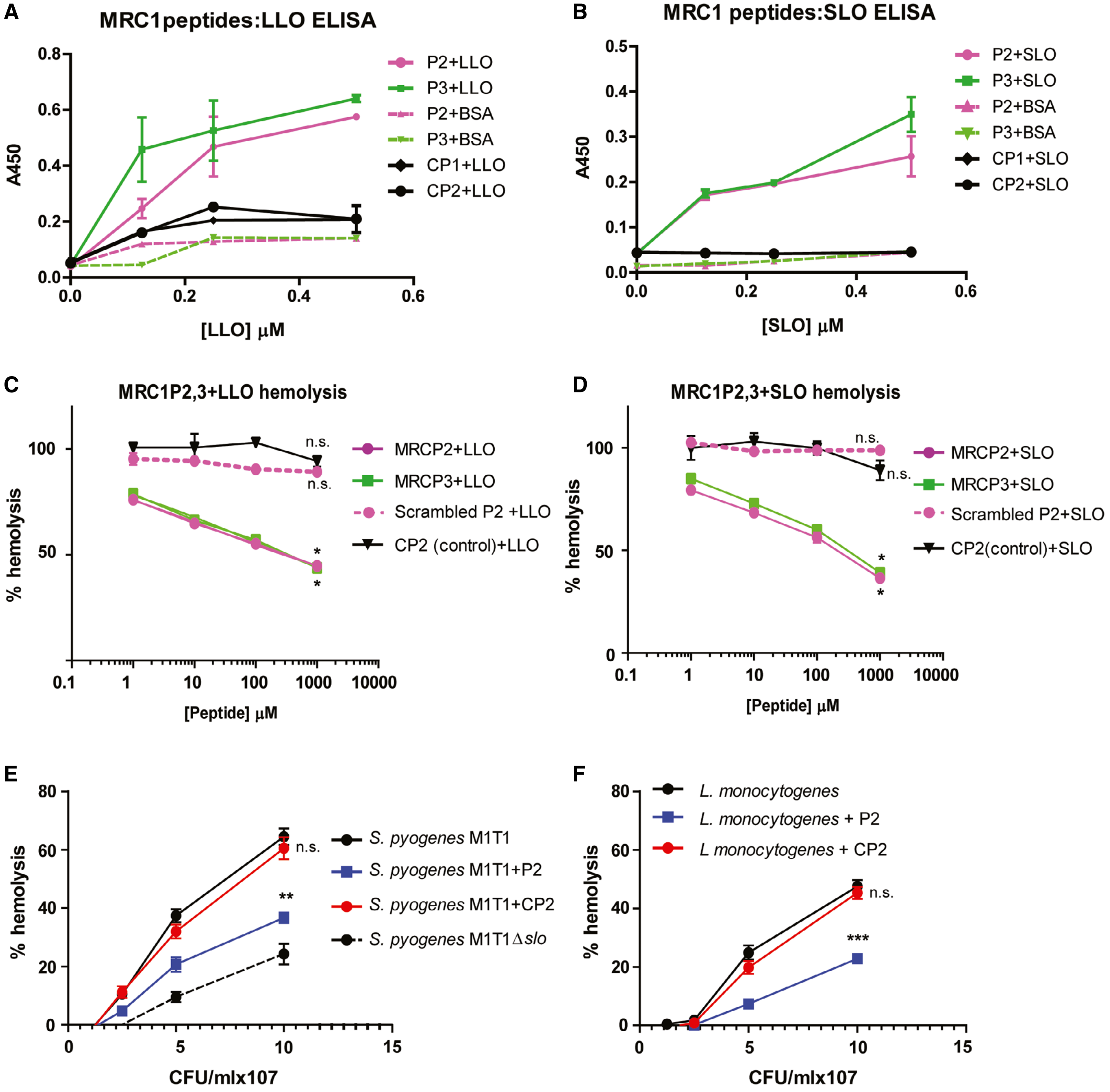

Figure EV3. Dose-dependent binding of peptides to LLO and SLO and inhibition of hemolysis and pro-inflammatory cytokine responses.

A, B ELISA showing the dose-dependent binding of plate-bound MRC-1 peptides P2, P3, and the control peptides CP1 and CP2 to (A) purified LLO and (B) SLO (0–0.5 µM). BSA was used as negative control to show the binding specificity. Data are mean ± s.e.m. from two independent experiments with triplicates.

C, D Hemolysis assay of 1 µg/ml purified (C) LLO and (D) SLO in the presence of increasing concentrations of MRC-1 peptides, P2, P3, scrambled P2, and control peptide CP2 (1–1,000 µM). Data are mean ± s.e.m. from 4 independent experiments. *$P < 0.05$ by one-way ANOVA with Dunnett's *post hoc* test for multiple comparisons. Exact *P* values are shown in Appendix Table S4.

E, F Hemolytic activity of (E) *S. pyogenes* type M1T1 and (F) *L. monocytogenes* in the presence of 100 µM peptide P2 and CP2. The isogenic SLO mutant strain was used as a negative control. Data are mean ± s.e.m. from three independent experiments. **$P < 0.01$ and ***$P < 0.001$ by one-way ANOVA with Bonferroni *post hoc* test for multiple comparisons. n.s. denotes not significant. Exact *P* values are shown in Appendix Table S4.

© 2020 The Authors

Figure EV4. The MRC-1 peptide P2 inhibits CDC-induced cytolysis and intracellular survival of bacteria in human DCs.

A, B (A) IL-12 and (B) TNF-α release by human THP-1 macrophages stimulated with purified PLY, LLO, or SLO (0.5 μg/ml) in the presence or absence of 100 μM peptides P2, P3, or control peptide CP2 for 18 h. Cholesterol (100 μM) was used as positive control to inhibit hemolysis. Data are mean ± s.e.m. from three independent experiments. $^{**}P < 0.01$, $^{***}P < 0.001$, and $^{****}P < 0.0001$ by two-way ANOVA with Bonferroni *post hoc* test for multiple comparisons. n.s. denotes not significant. Exact *P* values are shown in Appendix Table S4.

C The loss of the GFP signal intensity owing to cell death at 3 h post-stimulation of GFP-expressing 3D lung epithelial models (n = 3) was quantified relative to 1 h time point. Data are mean ± s.e.m. from two independent experiments with n = 3 model/condition. $^{*}P < 0.05$ by paired *t*-test. Exact *P* values are shown in Appendix Table S4.

D Cytotoxicity of the lung epithelial model stimulated with 1 μg/ml PLY in the presence or absence of 100 μM peptide P2 or the control peptide CP2 at 18 h. Data are mean ± s.e.m. from two independent experiments with n = 3 model/condition. $^{****}P < 0.0001$ by one-way ANOVA with Dunnett's *post hoc* test for multiple comparisons. Exact *P* values are shown in Appendix Table S4.

E, F (E) TNF-α and (F) IL-8 release by the lung epithelial model stimulated with 1 μg/ml PLY in the presence or absence of 100 μM peptide P2 or the control peptide CP2 at 18 h. $^{**}P < 0.01$ and $^{****}P < 0.0001$ by one-way ANOVA with Dunnett's *post hoc* test for multiple comparisons. Exact *P* values are shown in Appendix Table S4. Data are mean ± s.e.m. from two independent experiments with n = 3 model/condition.

G Immunofluorescence microscopy images showing intracellular pneumococci of type 4 or type 2 in infected human DCs treated or not with 100 μM peptide P2 or the control peptide CP2. Anti-PLY antibody was used as control to block PLY. DCs infected with D39 or T4 alone possessed intracellular bacteria (green) that co-localized with MRC-1 (red). Arrows indicate MRC-1 co-localized intracellular bacteria (yellow). DCs treated with peptide P2, but not CP2, were devoid of intracellular bacteria. Images are representative of two independent experiments. Scale bars, 10 μm.

© 2020 The Authors

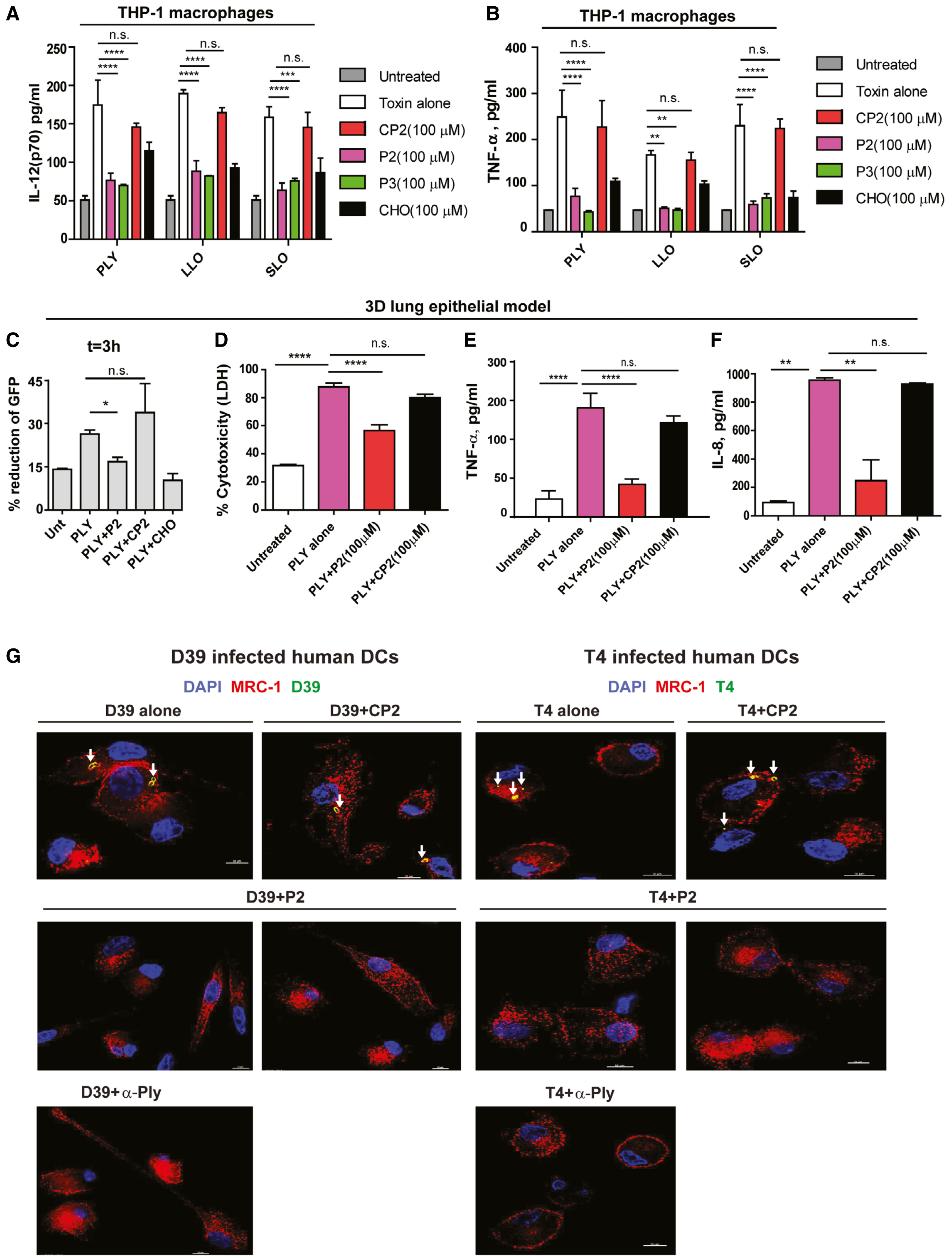

Figure EV4.

 © 2020 The Authors

Figure EV5. Effect of MRC-1 peptides on the intracellular bacterial localization in infected DCs.

A–C DCs were infected with (A) the PLY mutant strain T4RΔ*ply* only, or in the presence of 100 μM peptides, (B) P2 or (C) CP2 at MOI of 10 for 2 h. Immunofluorescence microscopy images show that peptide P2 had no effect on the co-localization of T4RΔ*ply* (green) which co-localized with LC3B (cyan), but not with MRC-1 (red). Images are representative of three independent experiments. Scale bars, 10 μm.

© 2020 The Authors

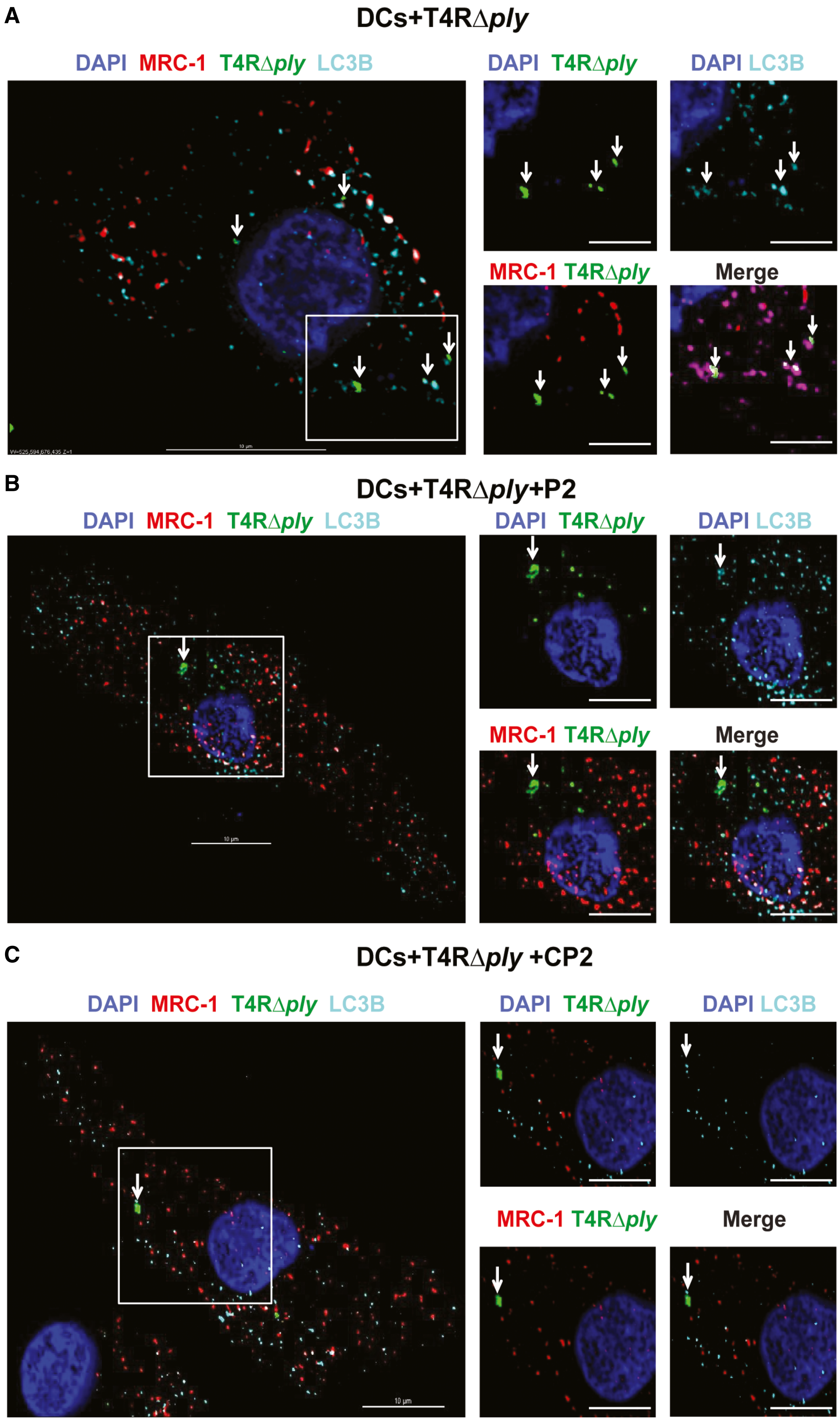

Figure EV5.

© 2020 The Authors
