# Supplementary material for: Mannose receptor‐derived peptides neutralize pore‐forming toxins and reduce inflammation and development of pneumococcal disease
Source: EMBO Mol Med. 2020 Sep 28;12(11):e12695. doi: 10.15252/emmm.202012695 (PMC7645366; doi:10.15252/emmm.202012695)
Supplement: Supplementary file 6 — Movie EV4 [file EMMM-12-e12695-s006.zip › Movie EV4.docx]

**Movies EV1-11**. Human THP-1 macrophages were loaded with live/dead reagent (2 μM Calcein AM and 4 μM Ethidium bromide) for 20 min at 37ºC and treated with 0.5 μg/ml PLY, LLO or SLO with or without 100 μM peptide P2 or the control peptide, CP2. Cells were imaged at 30s intervals for a total time of 20 min.

**Movie EV4.** Live-imaging of THP-1 macrophages treated with 0.5 μg/ml purified PLY in the presence of 100 μM control peptide CP2 for 20 min. Scale bar, 25 μm. The cells undergo membrane blebbing and stain orange due to uptake of propidium iodide, indicating cytolysis.
