# Supplementary material for: Mannose receptor‐derived peptides neutralize pore‐forming toxins and reduce inflammation and development of pneumococcal disease
Source: EMBO Mol Med. 2020 Sep 28;12(11):e12695. doi: 10.15252/emmm.202012695 (PMC7645366; doi:10.15252/emmm.202012695)
Supplement: Supplementary file 12 — Movie EV10 [file EMMM-12-e12695-s012.zip › Movie EV10.docx]

**Movies EV1-11**. Human THP-1 macrophages were loaded with live/dead reagent (2 μM Calcein AM and 4 μM Ethidium bromide) for 20 min at 37ºC and treated with 0.5 μg/ml PLY, LLO or SLO with or without 100 μM peptide P2 or the control peptide, CP2. Cells were imaged at 30s intervals for a total time of 20 min.

**Movie EV10.** Live-imaging of THP-1 macrophages treated with 0.5 μg/ml purified SLO in the presence of 100 μM control peptide CP2 for 20 min. Scale bar, 5 μm. The cells undergo membrane blebbing and stain orange due to uptake of propidium iodide, indicating cytolysis.
