# Supplementary figures and images for: Mannose receptor‐derived peptides neutralize pore‐forming toxins and reduce inflammation and development of pneumococcal disease
Source: EMBO Mol Med. 2020 Sep 28;12(11):e12695. doi: 10.15252/emmm.202012695 (PMC7645366; doi:10.15252/emmm.202012695)

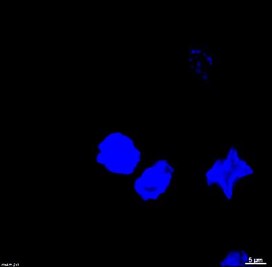

Supplement: Supplementary file 15 — Source Data Figure 1 [file EMMM-12-e12695-s014.zip › Fig. 1A DCs plus PLY_DAPI.jpg]

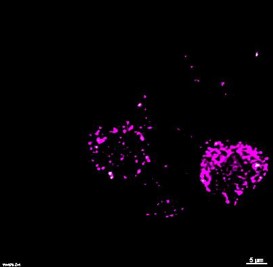

Supplement: Supplementary file 15 — Source Data Figure 1 [file EMMM-12-e12695-s014.zip › Fig. 1A DCs plus PLY_EEA-1.jpg]

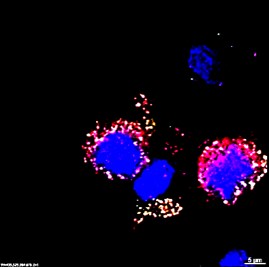

Supplement: Supplementary file 15 — Source Data Figure 1 [file EMMM-12-e12695-s014.zip › Fig. 1A DCs plus PLY_Merge.jpg]

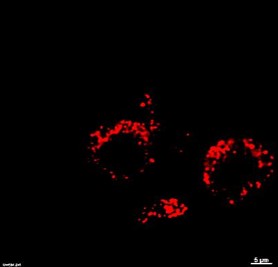

Supplement: Supplementary file 15 — Source Data Figure 1 [file EMMM-12-e12695-s014.zip › Fig. 1A DCs plus PLY_MRC1.jpg]

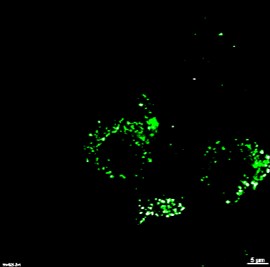

Supplement: Supplementary file 15 — Source Data Figure 1 [file EMMM-12-e12695-s014.zip › Fig. 1A DCs plus PLY_PLY.jpg]

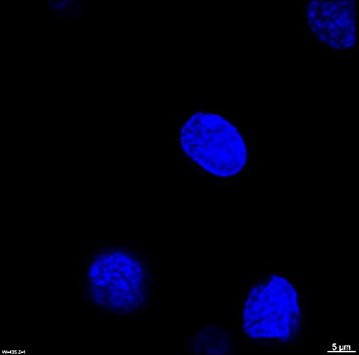

Supplement: Supplementary file 15 — Source Data Figure 1 [file EMMM-12-e12695-s014.zip › Fig. 1A untreated_DAPI.jpg]

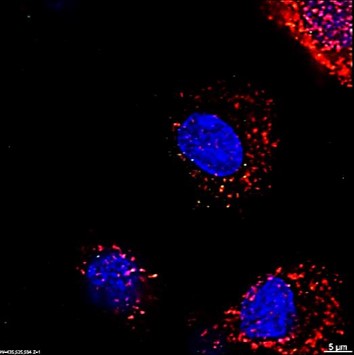

Supplement: Supplementary file 15 — Source Data Figure 1 [file EMMM-12-e12695-s014.zip › Fig. 1A untreated_Merge.jpg]

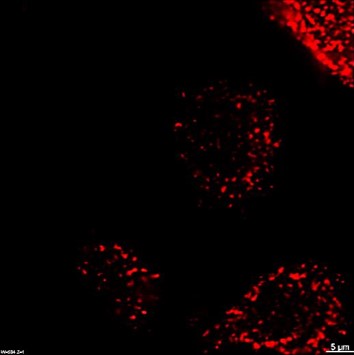

Supplement: Supplementary file 15 — Source Data Figure 1 [file EMMM-12-e12695-s014.zip › Fig. 1A untreated_MRC1.jpg]

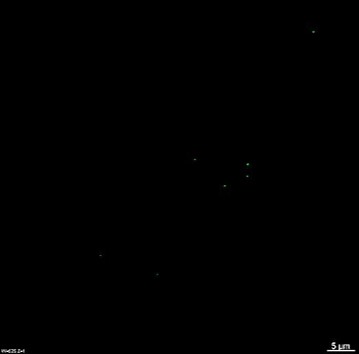

Supplement: Supplementary file 15 — Source Data Figure 1 [file EMMM-12-e12695-s014.zip › Fig. 1A untreated_PLY.jpg]

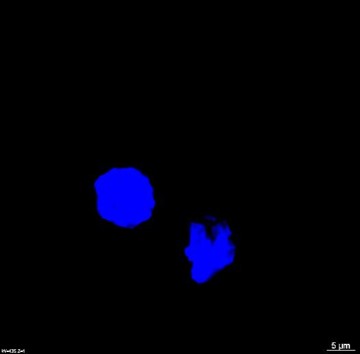

Supplement: Supplementary file 15 — Source Data Figure 1 [file EMMM-12-e12695-s014.zip › Fig. 1B DCs plus LLO_DAPI.jpg]

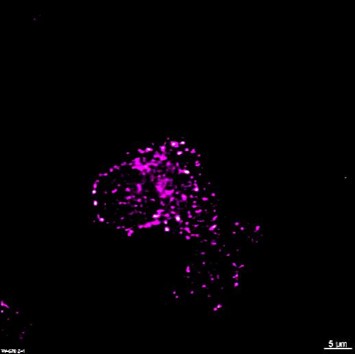

Supplement: Supplementary file 15 — Source Data Figure 1 [file EMMM-12-e12695-s014.zip › Fig. 1B DCs plus LLO_EEA-1.jpg]

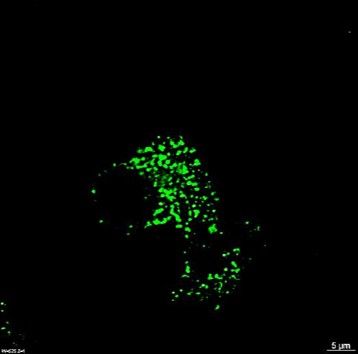

Supplement: Supplementary file 15 — Source Data Figure 1 [file EMMM-12-e12695-s014.zip › Fig. 1B DCs plus LLO_LLO.jpg]

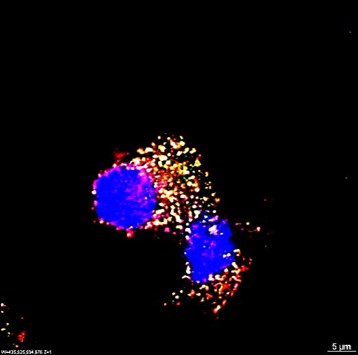

Supplement: Supplementary file 15 — Source Data Figure 1 [file EMMM-12-e12695-s014.zip › Fig. 1B DCs plus LLO_Merge.jpg]

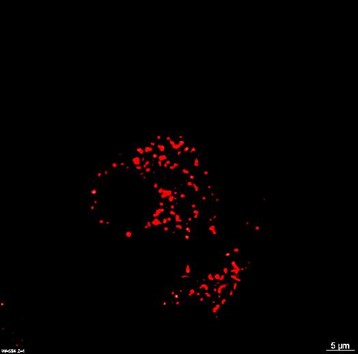

Supplement: Supplementary file 15 — Source Data Figure 1 [file EMMM-12-e12695-s014.zip › Fig. 1B DCs plus LLO_MRC1.jpg]

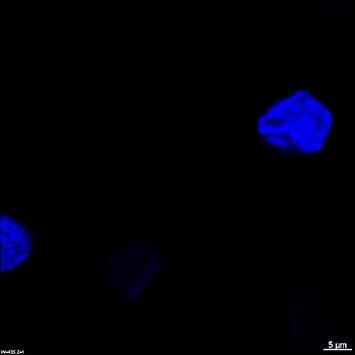

Supplement: Supplementary file 15 — Source Data Figure 1 [file EMMM-12-e12695-s014.zip › Fig. 1B untreated DAPI.jpg]

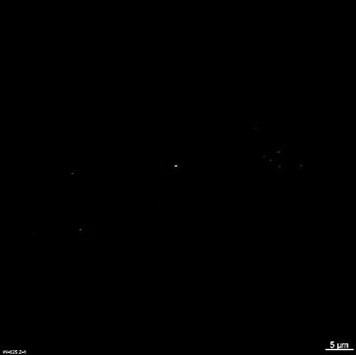

Supplement: Supplementary file 15 — Source Data Figure 1 [file EMMM-12-e12695-s014.zip › Fig. 1B untreated LLO.jpg]

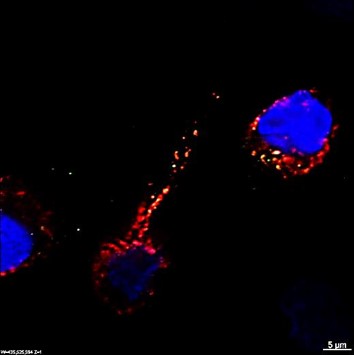

Supplement: Supplementary file 15 — Source Data Figure 1 [file EMMM-12-e12695-s014.zip › Fig. 1B untreated Merge.jpg]

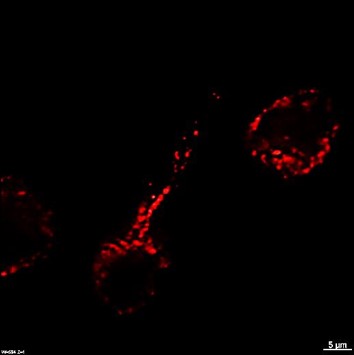

Supplement: Supplementary file 15 — Source Data Figure 1 [file EMMM-12-e12695-s014.zip › Fig. 1B untreated MRC1.jpg]

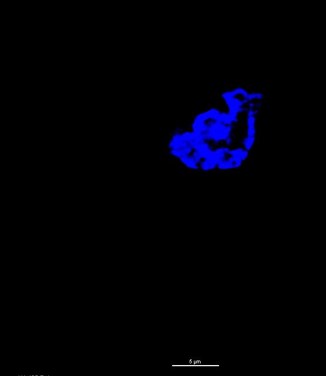

Supplement: Supplementary file 15 — Source Data Figure 1 [file EMMM-12-e12695-s014.zip › Fig. 1C DCs plus SLO_DAPI.jpg]

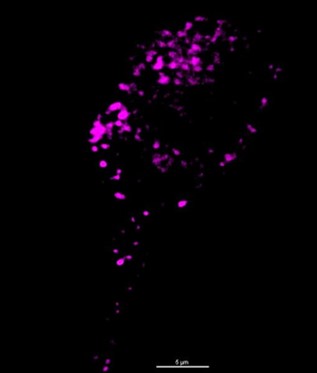

Supplement: Supplementary file 15 — Source Data Figure 1 [file EMMM-12-e12695-s014.zip › Fig. 1C DCs plus SLO_EEA-1.jpg]

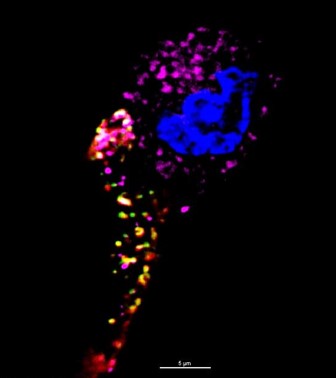

Supplement: Supplementary file 15 — Source Data Figure 1 [file EMMM-12-e12695-s014.zip › Fig. 1C DCs plus SLO_Merge.jpg]

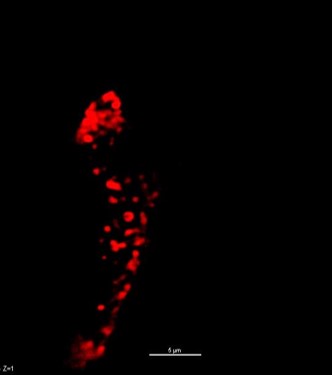

Supplement: Supplementary file 15 — Source Data Figure 1 [file EMMM-12-e12695-s014.zip › Fig. 1C DCs plus SLO_MRC1.jpg]

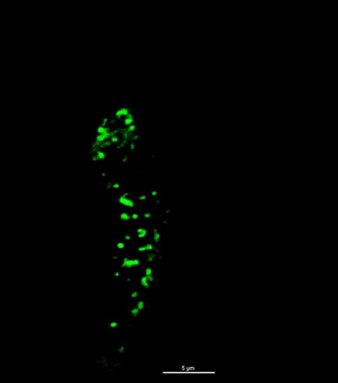

Supplement: Supplementary file 15 — Source Data Figure 1 [file EMMM-12-e12695-s014.zip › Fig. 1C DCs plus SLO_SLO.jpg]

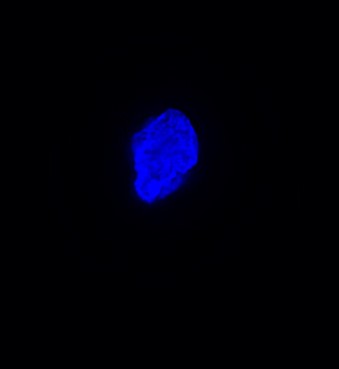

Supplement: Supplementary file 15 — Source Data Figure 1 [file EMMM-12-e12695-s014.zip › Fig. 1C untreated DAPI.jpg]

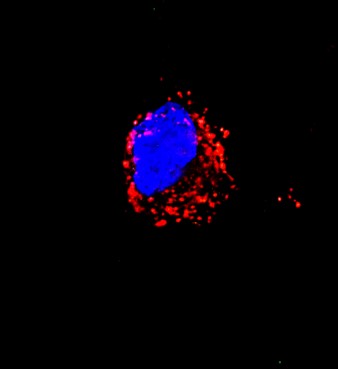

Supplement: Supplementary file 15 — Source Data Figure 1 [file EMMM-12-e12695-s014.zip › Fig. 1C untreated Merge.jpg]

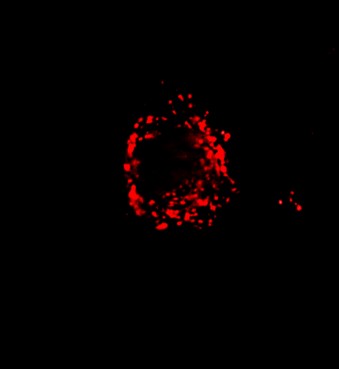

Supplement: Supplementary file 15 — Source Data Figure 1 [file EMMM-12-e12695-s014.zip › Fig. 1C untreated MRC1.jpg]

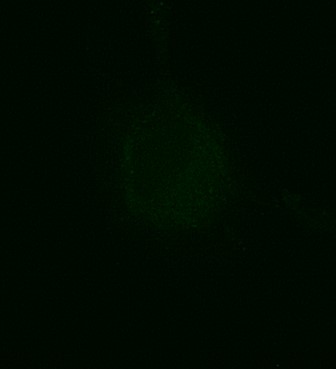

Supplement: Supplementary file 15 — Source Data Figure 1 [file EMMM-12-e12695-s014.zip › Fig. 1C untreated SLO.jpg]

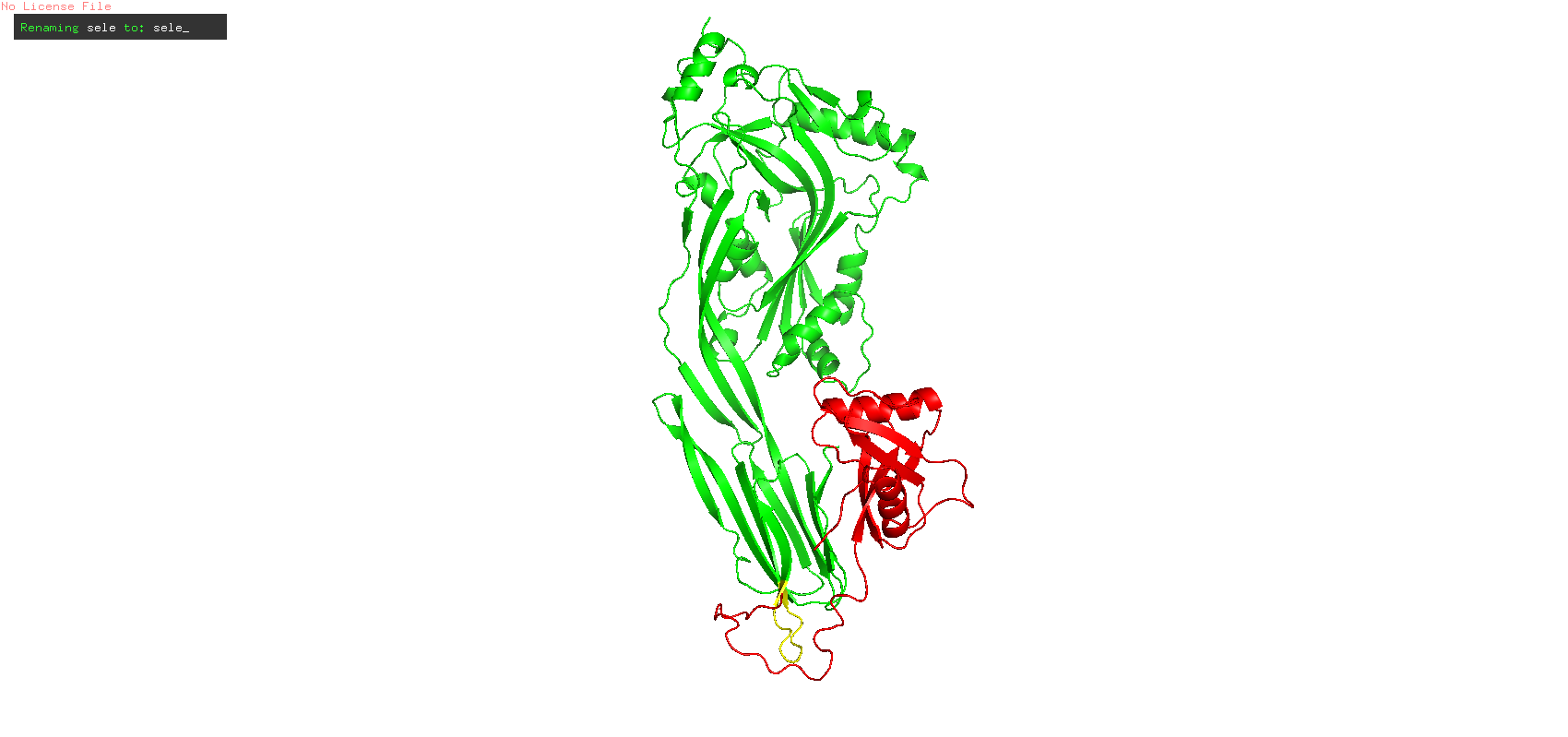

Supplement: Supplementary file 16 — Source Data Figure 2 [file EMMM-12-e12695-s015.zip › Fig. 2A.png]

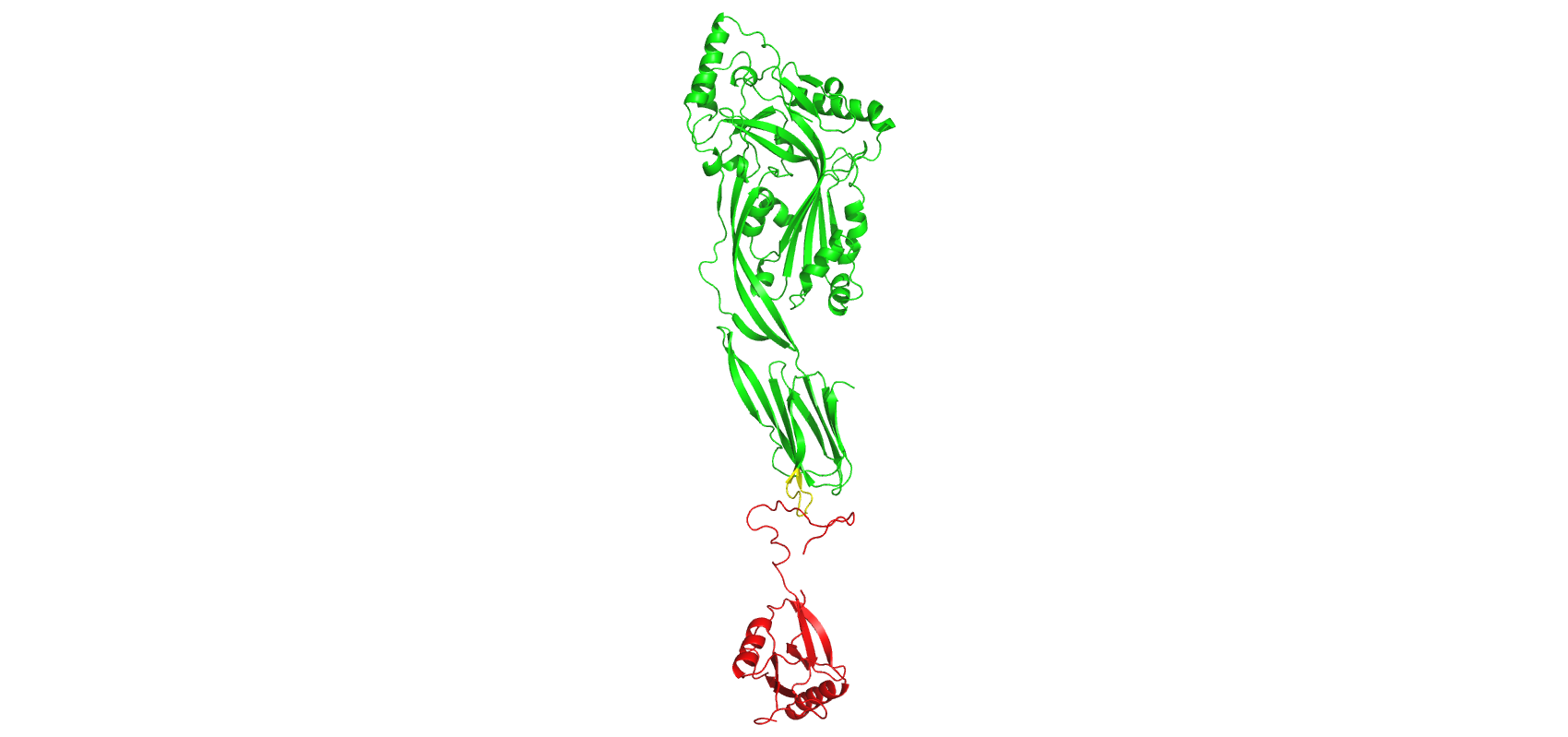

Supplement: Supplementary file 16 — Source Data Figure 2 [file EMMM-12-e12695-s015.zip › Fig. 2B.png]

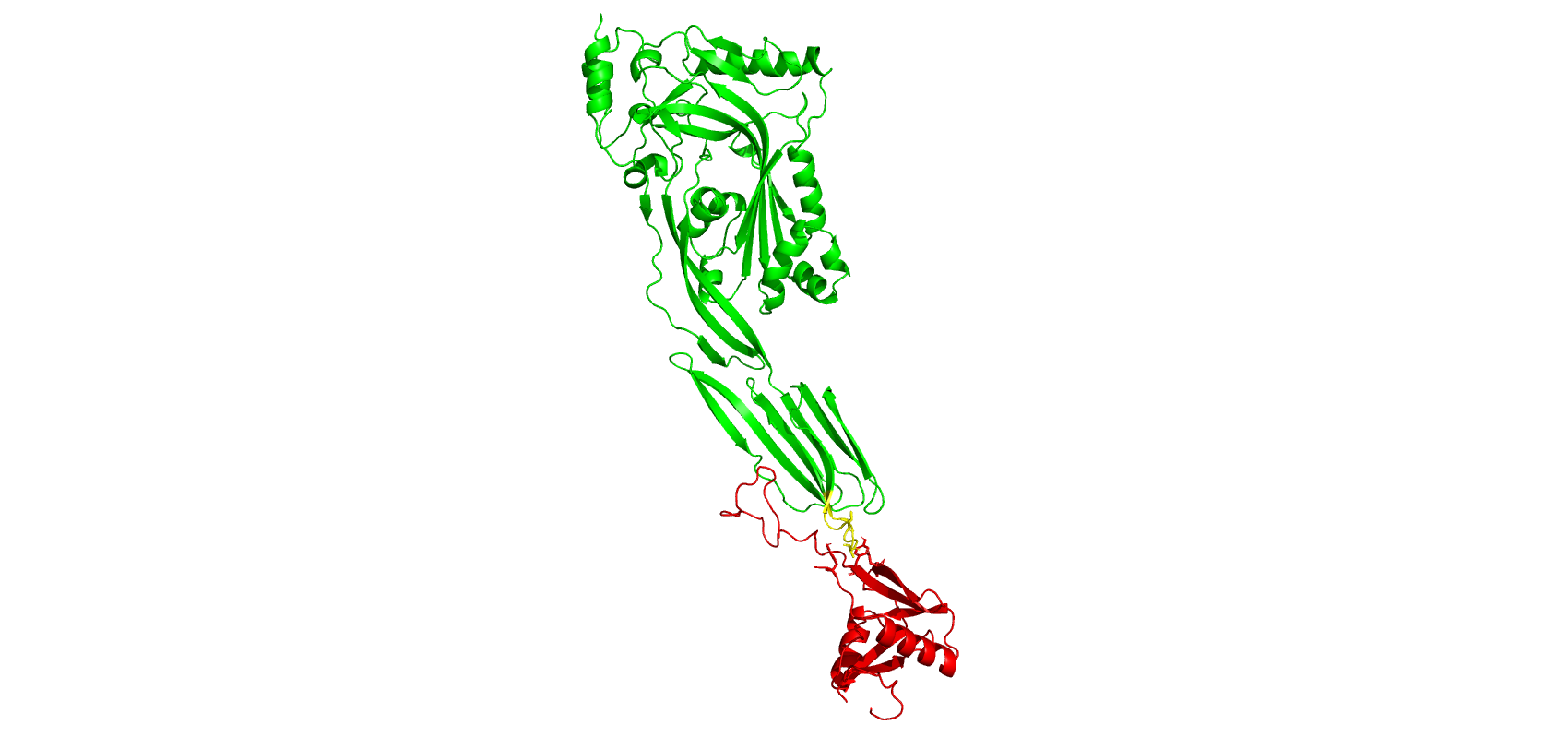

Supplement: Supplementary file 16 — Source Data Figure 2 [file EMMM-12-e12695-s015.zip › Fig. 2C.png]

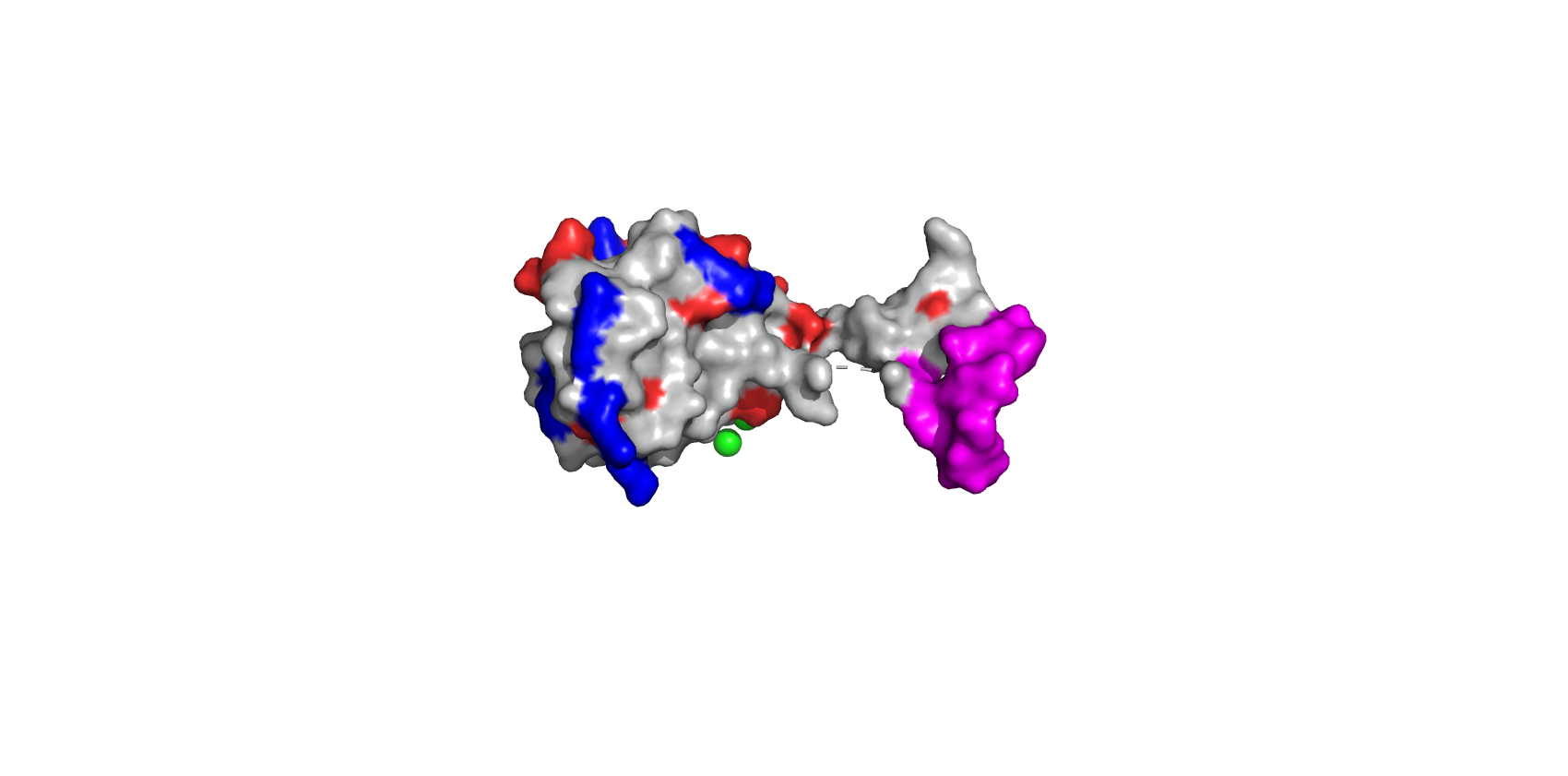

Supplement: Supplementary file 16 — Source Data Figure 2 [file EMMM-12-e12695-s015.zip › Fig. 2D.png]

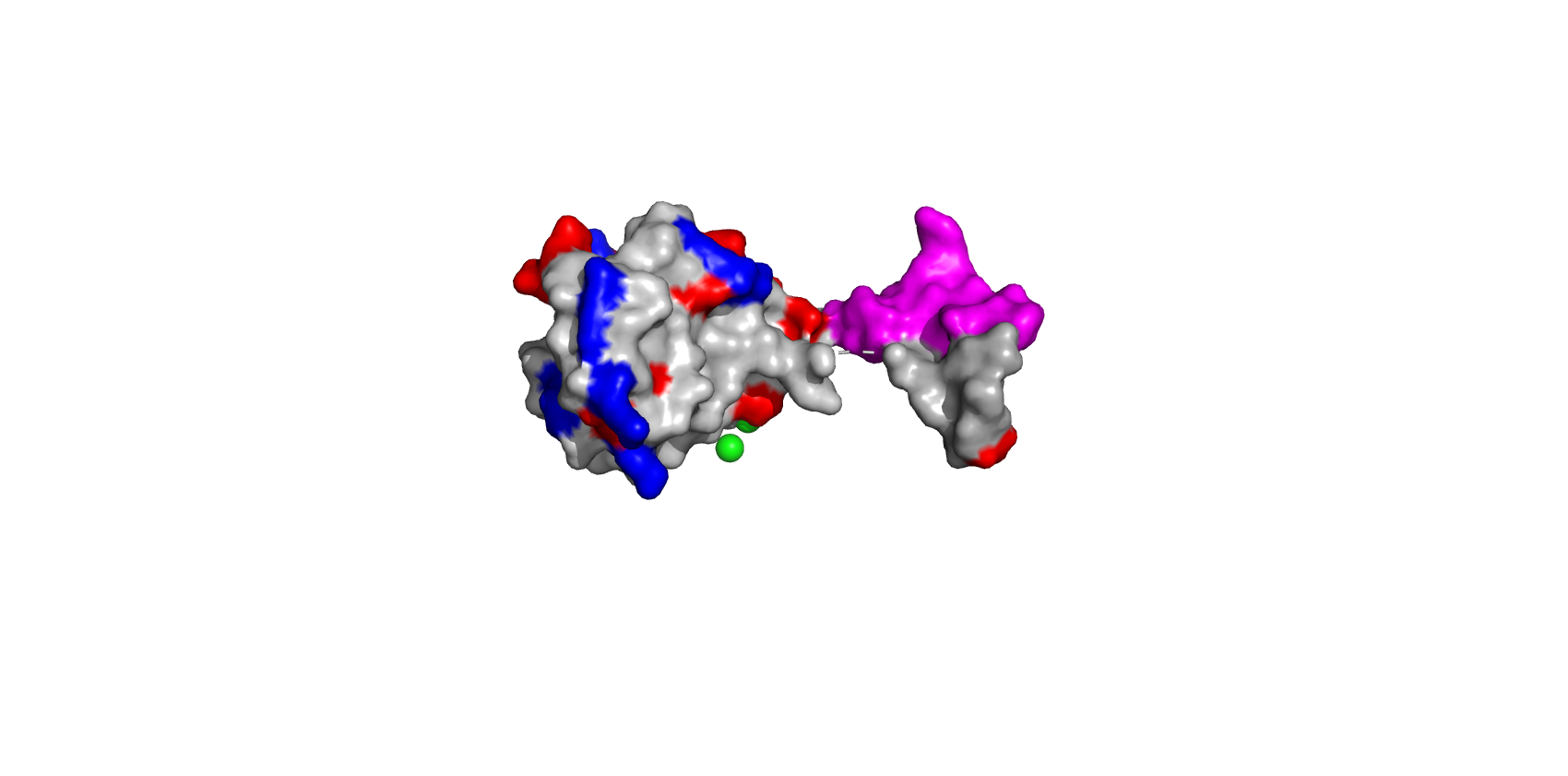

Supplement: Supplementary file 16 — Source Data Figure 2 [file EMMM-12-e12695-s015.zip › Fig. 2E.png]
